# Expert curation in UniProtKB: a case study on dealing with conflicting and erroneous data

**DOI:** 10.1093/database/bau016

**Published:** 2014-03-12

**Authors:** Sylvain Poux, Michele Magrane, Cecilia N. Arighi, Alan Bridge, Claire O’Donovan, Kati Laiho, The UniProt Consortium

**Affiliations:** ^1^SIB Swiss Institute of Bioinformatics, Centre Medical Universitaire, 1 rue Michel Servet, 1211 Geneva 4, Switzerland, ^2^European Molecular Biology Laboratory (EMBL), European Bioinformatics Institute, Wellcome Trust Genome Campus, Hinxton, Cambridge CB10 1SD, UK, ^3^Protein Information Resource, University of Delaware, 15 Innovation Way, Suite 205, Newark, DE 19711, USA and ^4^Protein Information Resource, Georgetown University Medical Center, 3300 Whitehaven Street North West, Suite 1200, Washington, DC 20007, USA

## Abstract

UniProtKB/Swiss-Prot provides expert curation with information extracted from literature and curator-evaluated computational analysis. As knowledgebases continue to play an increasingly important role in scientific research, a number of studies have evaluated their accuracy and revealed various errors. While some are curation errors, others are the result of incorrect information published in the scientific literature. By taking the example of sirtuin-5, a complex annotation case, we will describe the curation procedure of UniProtKB/Swiss-Prot and detail how we report conflicting information in the database. We will demonstrate the importance of collaboration between resources to ensure curation consistency and the value of contributions from the user community in helping maintain error-free resources.

**Database URL:** www.uniprot.org

## Introduction

Manual curation is a time-consuming and expensive process, but it undoubtedly adds great value to resources such as the UniProt Knowledgebase (UniProtKB). UniProtKB comprises two sections, UniProtKB/Swiss-Prot, the reviewed section containing manually curated records with information extracted from the literature and curator-evaluated computational analysis, and UniProtKB/TrEMBL, the unreviewed section with automatically annotated records (1).

Knowledgebases play an increasingly important role in aiding scientific research and discovery by providing data in easily accessible formats. A number of recent reports have raised the question of the reliability of these resources and have highlighted the presence of errors and/or incomplete information contained in databases and their consequences. For example, a paper published by the Babbitt group investigated the misannotation levels for molecular function in four public protein sequence databases for a set of 37 enzyme families for which extensive experimental information is available and concluded that the level of erroneous annotation was much higher in automatically annotated databases than in manually curated resources (2). The quality of Gene Ontology (GO) electronic annotations and their limitations have also been assessed (3), demonstrating significant variability among inference methods, types of annotations and species while showing continued improvement of these annotations. A recent article reported how an annotation error in a UniProtKB/Swiss-Prot entry, due to the interpretation of an incomplete functional characterization paper, persisted for 20 years and was disseminated to other databases (4).

While these papers describe curation errors, they do not examine the curation process in different knowledgebases. A good understanding of the annotation content of a database and how it is generated is required for its correct usage. This is exemplified by a paper published in *PLoS Computional Biology* that concluded that paralogous genes within the mouse or human genomes are more functionally similar on average than orthologous genes between these genomes (5). This analysis, which was based on experimental GO annotation, suffered from an incomplete understanding of the GO annotations that biased the results. As demonstrated later by another group (6), differences in annotations between pairs of orthologous genes reflect complementarity in experimental approaches rather than differences in biological function, with some types of experiments being performed in one organism and not in the other. Moreover, GO annotations are frequently incomplete, resulting in annotation differences even in the absence of functional differences.

It is clear that knowledgebases contain a small proportion of errors, and some of them are due to the misinterpretation of data by curators, but contradictory or incorrect results in the scientific literature highly complicate the curation task, and curators frequently have to attempt to reconcile conflicting data from different publications. A recent article published in *The Economist* (Trouble at the lab; www.economist.com/news/briefing/21588057-scientists-think-science-self-correcting-alarming-degree-it-not-trouble) showed how the growing number of errors found in the scientific literature is reaching such an alarming level that science self-correction is not possible anymore. The article cites a number of studies that have tried to reproduce results found in the literature without success. In an article in *Nature*, for example, scientists from Amgen reported that they could only reproduce 6 of 53 studies considered as landmarks in the field of cancer research (7). Another publication, from researchers at Bayer HealthCare, reported that they could successfully reproduce results in only 25% of the cases (8).

By taking the example of sirtuin-5 (SIRT5), a complex annotation case within what was considered to be a well-characterized protein family, we will describe how expert curation is performed in UniProtKB/Swiss-Prot. SIRT5 belongs to the class III subfamily of sirtuins, a subfamily conserved from human to bacteria. Although protein deacetylase activity was initially reported for SIRT5 in humans and mice, recent data have shed new light on its activity by showing that it acts instead as a protein deacylase. We will detail how we report conflicting results found in the literature and describe collaborations with other resources. We will also show how curating information facilitates its dissemination as well as its subsequent use in automatic annotation and function-prediction systems by establishing a pipeline where manual and automatic annotation processes are linked. We believe that a better understanding of the manual curation process is a prerequisite for the correct interpretation and usage of the content of knowledgebases.

## The SIRT5 case

Sirtuins, also called Sir2 proteins, are NAD-dependent deacetylases that regulate important biological processes. The name ‘Sir2’ comes from the yeast ‘silent information regulator 2’ gene, a gene involved in a range of processes including transcriptional repression. Sirtuins belong to a family of evolutionary conserved proteins occurring in all kingdoms. Mammals have seven sirtuins, SIRT1–SIRT7 (9). Robust deacetylase activity has been demonstrated for mammalian SIRT1–SIRT3, and the annotation concerning this function has been propagated to other paralogs on the basis of their sequence similarity. However, so far, SIRT4–SIRT7 have shown only very weak, if any, deacetylase activity (10). Recent data on SIRT5, a member of the class III subfamily, a subfamily conserved from human to bacteria, illustrate how new functions continue to be discovered within what are thought to be well-characterized protein families.

In an initial report, the human SIRT5 protein was shown to have protein deacetylase activity *in vitro* (11). Later, it was shown to act as a key regulator of the urea cycle by activating the CPS1 enzyme *in vivo* in mice (12). The authors showed that SIRT5 had the ability to deacetylate CPS1 *in vitro* and concluded that SIRT5 activates CPS1 via deacetylase activity. However, they did not show that the activation of CPS1 is the result of protein deacetylation. Nevertheless, the deacetylase activity of SIRT5 has been accepted for many years.

A major breakthrough in the field came from two independent studies in late 2011 (10, 13). The crystal structure of the human SIRT5 protein showed that the pocket used by SIRT2 to host acetyl groups appears to be much larger in SIRT5, large enough to host a negatively charged acyl group instead (10). In cells, the most common acyl-CoA molecules with a carboxylate group are malonyl-CoA and succinyl-CoA, so malonyl- and succinyl-peptides were produced and tested as substrates for SIRT5. SIRT5 displayed strong activity toward these peptides and was able to catalyze their hydrolysis, proving its demalonylase and desuccinylase functions. Lysine malonylation and succinylation represent two new previously unknown post-translational modifications (PTMs), and a number of proteins that are malonylated and/or succinylated *in vivo* have been identified in the same publications (10, 13).

Previous data published on SIRT5 were then reinvestigated (12) and its role in the activation of CPS1 studied. It was confirmed that SIRT5 can activate CPS1: in SIRT5-knockout mice, CPS1 is not activated following fasting, leading to elevated blood ammonia levels. It was also demonstrated that CPS1 is succinylated at specific residues *in vivo*. Finally, acetylation levels were shown to be unchanged in SIRT5-knockout mice while succinylation is strongly increased, definitively proving that SIRT5 hydrolyzes succinyl groups and not acetyl groups. The following section will demonstrate how such information is reported in UniProtKB/Swiss-Prot.

## Expert curation in UniProtKB/Swiss-Prot

Expert curation in UniProtKB/Swiss-Prot follows a well-defined process to ensure that all records are handled in a consistent manner (see www.uniprot.org/faq/45 for a more detailed description of the process). It includes manual verification of each protein sequence as well as a critical review of experimental data from the literature and predicted data from a range of sequence analysis tools (14). Publications are read in detail and fully curated. Curators assimilate all the information from various sources, reconcile any conflicting results and compile the data into a concise but comprehensive report, which provides a complete overview of the information available about a particular protein (14). The UniProt curation team consists of experienced, generally PhD-level, biologists or biochemists with a strong background in wet lab research.

To provide high-quality in-depth experimental annotation, the choice of publications to use is critical. We select publications to curate according to well-established criteria. The following categories of literature are prioritized during the manual curation process: publications with (i) a high impact in the scientific community that contain functional data for previously uncharacterized proteins, (ii) new 3D-structural information, (iii) enzymatic reactions that may complete the annotation of known metabolic pathways or networks, (iv) PTMs and their consequences, (v) novel splice variants and (vi) disease-causing variants as well as polymorphisms. One of the main challenges of manual curation is to capture the maximum amount of available information while recognizing that it is impossible to curate all publications. We do not aim to curate all published papers and instead select a representative subset to provide a complete overview of available information.

**Figure 1.**
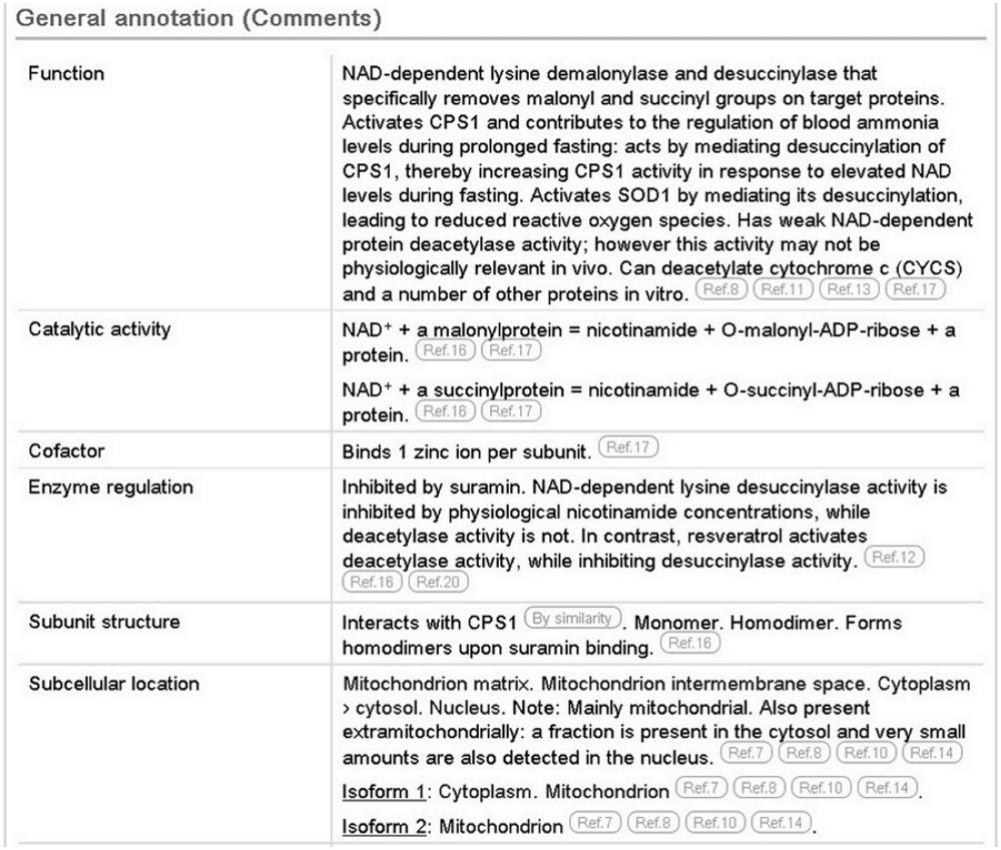
Screenshot of the general annotation section of the human SIRT5 entry (UniProtKB Q9NXA8, http://www.uniprot.org/uniprot/Q9NXA8).

In the case of SIRT5 (UniProtKB Q9NXA8), the literature has been used to provide information about its demalonylase and desuccinylase activities and to highlight the importance of desuccinylation in the regulation of the urea cycle. We report the deacetylase activity, but indicate that this activity may not exist *in vivo* (Figure 1, function subsection).

**Figure 2.**
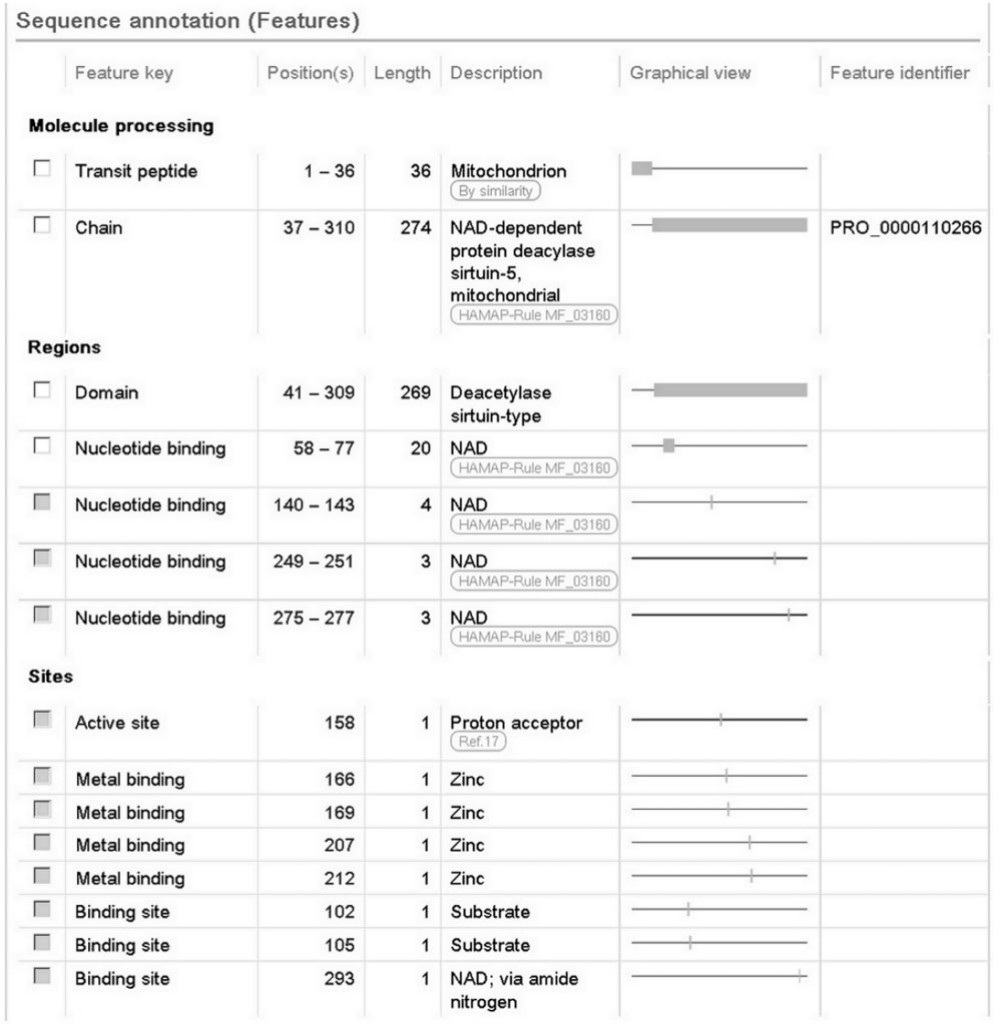
Screenshot of the sequence annotation section of the human SIRT5 entry (UniProtKB Q9NXA8, http://www.uniprot.org/uniprot/Q9NXA8).

We also report a wealth of additional experimentally determined information including the subcellular location, enzyme regulation, catalytic activity and cofactor. The 3D structure described earlier provides information about the subunit structure and shows the interactions of the protein with NAD, zinc and substrate. The positions of these binding sites are annotated (Figure 2), using information from the Protein Data Bank (PDB) in combination with author information extracted from the paper. All information added during the manual annotation process is linked to its original source so that users can trace the origin of each piece of information and evaluate it (Figure 1). UniProtKB data is structured in a highly standardized way using controlled vocabularies to simplify data access for users and data retrieval by computer programs, and it aids in use of our data by other databases. This is described in more detail in the next section.

In many cases, experiments are performed in a range of different strain and species backgrounds for a single protein. In the case of SIRT5, the 3D experiments and enzyme studies were done with the human protein, whereas knockout experiments were performed in mice. This information has been reported in UniProtKB Q9NXA8 and Q8K2C6, respectively.

Identification of the malonylated and succinylated proteins was performed in cows. We therefore report the post-translational lysine malonylation and succinylation modifications sites in the corresponding bovine entries: UniProtKB P12344, Q29RK1, P00366, Q2KIE6, Q32LG3 and P00586. When the effect of lysine malonylation and succinylation is known, as in CPS1, it is indicated. In the mouse CPS1 entry (UniProtKB Q8C196), we have also added a CAUTION comment to warn users that the deacetylation initially reported as being carried out by SIRT5 does not occur *in vivo*.

As mentioned before, SIRT5 is evolutionary conserved and homologous proteins are present in different kingdoms. Characterization of a remote SIRT5 ortholog in *Plasmodium falciparum* (Q8IE47) confirmed the deacylase activity (15). However, this protein lacks the conserved residues that bind to malonylated and succinylated substrates and displays hydrolase activity toward different acyl groups and removes medium- and long-chain fatty acids, illustrating the need for careful propagation to homologous proteins.

UniProt entries are regularly updated as new information becomes available and SIRT5 entries have been updated a number of times during the past 2 years. It is interesting to note that, while the deacylase activity of SIRT5 has been clearly proven, a number of papers still publish on the deacetylase activity of SIRT5 based on *in vitro* and/or indirect analysis (16, 17). We have also checked whether enzymes that mediate the succinylation and malonylation modifications have been identified. However, it does not seem to be the case at this time.

## Collaboration with other resources

UniProt actively collaborates with and leverages the work of complementary curated resources to facilitate consistency and data exchange and to ensure that curation efforts are not duplicated. We have established a number of collaborations in the field of sequence curation with other groups such as Ensembl (18) and RefSeq (19) and with organism-specific databases to ensure that users are provided with a consistent set of protein sequences across these resources. Such collaborative efforts also exist in the area of literature curation and frequent exchanges take place between UniProtKB curators and curators at other resources. A good example is provided by our collaboration with the Saccharomyces Genome Database (20) to solve discrepancies that exist between our Enzyme Commission numbers.

The update of the SIRT5 records involved the use of and contribution to a number of controlled vocabularies, the use of standardized vocabularies being essential to the task of organizing knowledge for subsequent retrieval. These are outlined later and demonstrate the importance of standardized vocabularies in the curation process and the improvements that UniProt has made to these.

We actively collaborate with resources in the area of nomenclature. Protein names are established according to naming guidelines we have developed: we assign a ‘Recommended name’ to the protein, which is, as far as possible, unique and attributed to orthologs (http://www.uniprot.org/docs/nameprot). These guidelines have promoted the use of correct and standardized protein names and are reused by a number of databases and institutions; the American Society for Microbiology and the Joint Commission on Biochemical Nomenclature have discussed these guidelines and endorsed them, and our protein names are used by the NCBI (21). We report nomenclature found in publications and names are assigned in collaboration with Model Organism Databases (MODs) and nomenclature committees. For SIRT5, we assigned a new ‘Recommended name’ and also report gene names as recommended by the various MODs to ensure consistent nomenclature between resources.

UniProt is a major contributor to the GO (22), a major bioinformatics initiative that aims to standardize the representation of gene and gene product attributes across species and databases, and manual curation of GO terms based on experimental data from the literature is part of the UniProt curation process. We have a close collaboration with the GO editors who are responsible for ontology developments, and propose suggestions for new terms or correction of existing terms when required. For SIRT5, we proposed four new terms describing the new enzymatic activities and manually curated these terms. These new terms have been reused by other databases such as InterPro. To report conflicting results regarding the protein deacetylase activity of SIRT5, we also added the NOT qualifier to the ‘GO:0006476 protein deacetylation’ term to indicate that this has been refuted in recent reports. This qualifier has been introduced by the GO Consortium specifically to indicate that a gene product is not associated with a GO term when an association could be expected from previous literature or automated methods. Further details can be found at www.geneontology.org/GO.annotation.conventions.shtml#not.

We use other controlled vocabularies in a number of annotation fields and collaborate with external resources to contribute to and maintain these vocabularies. For example, every described PTM is associated with a controlled vocabulary established in collaboration with the RESID database (23) and is also linked to the corresponding term in PSI-MOD (24). For SIRT5, we created two new features to describe lysine malonylation and succinylation modifications.

The catalytic activity annotation field follows the recommendations of the Nomenclature Committee of the International Union of Biochemistry and Molecular Biology, and we actively participate in the creation of new Enzyme Commission numbers (25). The new reaction described for SIRT5 has been submitted to the International Union of Biochemistry and Molecular Biology.

Such mutually beneficial collaborations are essential because they help to improve the content of knowledgebases by reducing the number of errors and ensuring consistency across resources.

## From manual to automatic annotation

How do we propagate experimentally determined information to uncharacterized homologous proteins? Great care must be taken when propagating information to related proteins to avoid the generation of errors. The case of the *P. falciparum* homolog described earlier demonstrates that not all information can be safely propagated, as, in this case, we cannot specify the precise acyl groups hydrolyzed by homologs. To safely propagate information, we have developed the UniRule system, which uses manually curated annotation rules to enrich uncharacterized proteins in the unreviewed UniProtKB/TrEMBL section. The UniRule system, which will be the subject of a forthcoming publication by the UniProt consortium, includes annotation rules derived from HAMAP, a collection of manually curated family profiles, which are used to determine family membership of protein sequences (26). Family profiles are linked to manually curated annotation rules, which specify the annotation that can be applied to members of the protein family, and which include additional control statements that supervise the propagation of this annotation to member sequences in UniProtKB/TrEMBL (26). Numerous conditions must be satisfied for annotation propagation to proceed, ensuring high specificity of the annotations produced. This design feature is intended to reduce the likelihood of over-annotation, a relatively common error in some automated pipelines.

We generated a family rule for the SIRT5 family, based on expert curation in characterized members, disseminating high-quality annotation to members of the family in more than 3000 UniProtKB/TrEMBL entries (see http://hamap.expasy.org/profile/MF_01121). Based on the results obtained with the Plasmodium homologous protein (15), the demalonylase and desuccinylase activities are only propagated to proteins that contain the conserved residues that bind malonylated and succinylated substrates, while other proteins are described as deacylases. Last but not least, the rule only applies to members of the class III subfamily and paralogous sirtuins are not affected by the rule, limiting the number of false positives.

## Discussion

The selection of relevant and accurate literature is a key factor in the manual curation process. From this point of view, the SIRT5 example is striking because the curation of three key publications generated the complete reannotation of the SIRT5 protein in human and mouse, the correction of published erroneous information in these records, the annotation of the *P. falciparum* homolog, the addition of PTM sites to 28 different entries and the creation of a number of new controlled vocabulary terms in collaboration with other groups including four new GO terms, two new PTMs and a new catalytic activity. Last but not least, experimental information for these three proteins was used to create a rule for generation of automatic annotation.

Feedback from our user community is an essential element in helping to ensure data quality and prevent propagation of erroneous information, and update requests from users are dealt with as a priority. From this perspective, while we acknowledge the work of Percudani *et al.* (4) in detecting annotation errors in two UniProtKB/Swiss-Prot entries, we regret that they did not send any feedback before publication, in contradiction to what was claimed in the paper. This illustrates the unfortunate situation where scientists do not provide feedback concerning annotation errors to databases at the time of discovery, perhaps because they intend to publish them and overlook informing the concerned databases following publication or because they do not wish to devote time to this valuable exercise. This is regrettable because active collaboration with the user community is an essential prerequisite to avoiding errors as well as discrepancies between resources.

It is always difficult to handle conflicting or erroneous information. However, as illustrated with SIRT5, reading and curating a number of publications from different groups in different organisms helps to resolve conflicting issues and provides curators with a general overview of the state of research in the field. Moreover, it ensures maximal efficiency when curating groups of related proteins by reducing the time of curation, as curators already have the background knowledge in the field. It is important to note that a publication should always be curated in its background context: when information is in contradiction with previous reports, it should be clearly mentioned in the entry. Similarly, when previous research turns out to be erroneous, it should be stated clearly in databases to avoid confusion.

It is much more difficult to detect erroneous information when there is no publication to contradict data. UniProt curators frequently detect and report certain types of inconsistencies, such as enzymes lacking active sites reported to have activity, but many errors are difficult to identify. Indeed, many conclusions found in publications are based on indirect assumption rather than on definitive proof. The case of SIRT5 is again exemplary because it suggests that some sites previously thought to be acetylated may be malonylated and/or succinylated as well. It is, however, impossible to reinvestigate such sites in the absence of further evidence. Moreover, while the SIRT5 deacylase activity has been proven, some publications still report the deacetylase activity, generating additional erroneous data and contributing to the confusion that exists in the literature related to the function of this protein (16, 17). For example, Nakamura *et al.* (16) recently published that SIRT5 mediates protein deacetylation based on *in vitro* assays and an anti-acetylated lysine antibody, recognizing that their antibody cross-reacts with some acylated lysines. This example confirms again the importance of reading a range of publications in a particular area rather than isolated papers to ensure both efficiency and critical analysis of the data. While our initial reaction was to omit this publication, we eventually decided to include it to inform users that there is a bias in the analysis and to mitigate the conclusions of the article.

Although it is relatively easy to track papers that have been retracted and to remove associated annotations from databases, the number of retractions is very small because only 0.2% of published papers are retracted annually, whereas most papers with serious flaws remain (Trouble at the lab; www.economist.com/news/briefing/21588057-scientists-think-science-self-correcting-alarming-degree-it-not-trouble). Conscious of the problem, *Nature* and the other *Nature* research journals recently introduced a checklist to prompt authors to disclose technical and statistical information in their submissions, and to encourage referees to consider aspects important for research reproducibility (27). Such initiatives should be warmly acknowledged, as they promote the use of standards that could help the work of curators in the future.

**Figure 3.**
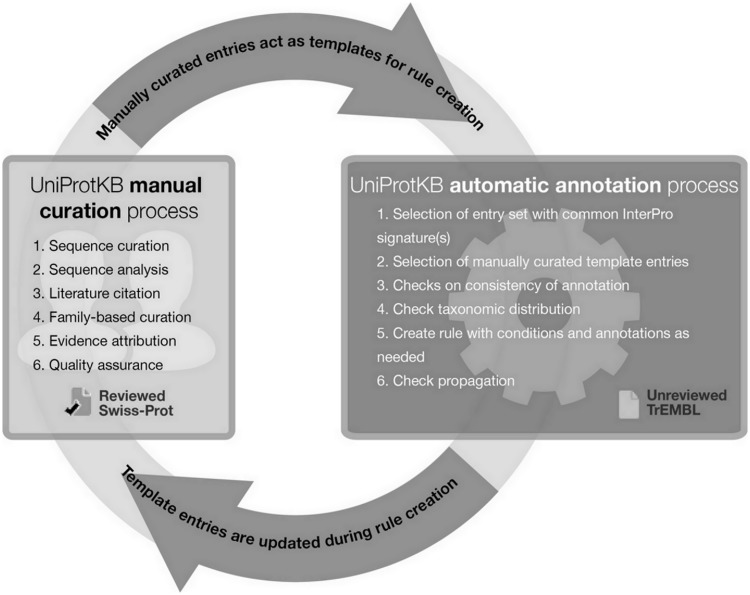
UniProtKB manual and automatic biocuration processes.

In addition to describing the role and importance of literature-based curation, we show a concrete example of how manual curation acts as the basis for automatic annotation via the creation of a family rule for class III sirtuins and its application in the unreviewed section of UniProtKB, UniProtKB/TrEMBL. By establishing a pipeline where manual and automatic annotation processes are linked (Figure 3), we ensure maximum efficiency without compromising the quality of the annotations produced. Such systems provide one possible answer to the concerns addressed by Schnoes *et al.* (2) regarding the high number of misannotations in automatically curated databases. Since the publication of their paper, the automated annotation pipeline has been improved by the addition of a large number of new and updated manually curated rules, generating high-quality annotation with the necessary granularity. Newly sequenced proteomes that enter the database are automatically annotated using UniRules, and are reannotated when template proteins of the family are manually updated (26).
